# Spatiotemporal dynamics of confirmed case distribution during the COVID-19 pandemic in China: data comparison between 2020/04–2020/08 and 2021/04–2021/08

**DOI:** 10.1038/s41598-023-39139-w

**Published:** 2023-07-23

**Authors:** Lin-Tao Fu, Zhong-Ling Qu, Xin Zeng, Liang-Zhi Li, Run Lan, Yu Zhou

**Affiliations:** School of Architecture and Civil Engineering, Chengdu University, Chengdu, 610106 China

**Keywords:** Epidemiology, Health policy, Epidemiology

## Abstract

The COVID-19 pandemic across Chinese mainland was gradually stabilized at a low level with sporadic outbreaks, before the emergence of Omicron variant. Apart from non-pharmacological interventions (NPIs), COVID-19 vaccine has also been implemented to prevent and control the pandemic since early 2021. Although many aspects have been focused, the change of the spatiotemporal distribution of COVID-19 epidemic across Chinese mainland responding to the change of prevention and control measures were less concerned. Here, we collected the confirmed case data (including domestic cases and overseas imported cases) across Chinese mainland during both 2020/04–2020/08 and 2021/04–2021/08, and then conducted a preliminary data comparison on the spatiotemporal distribution of confirmed cases during the identical period between the two years. Distribution patterns were evaluated both qualitatively by classification method and quantitatively through employing coefficient of variation. Results revealed significant differences in the homogeneity of spatiotemporal distributions of imported or domestic cases between the two years, indicating that the important effect of the adjustment of prevention and control measures on the epidemic evolution. The findings here enriched our practical experience of COVID-19 prevention and control. And, the collected data here might be helpful for improving or verifying spatiotemporally dynamic models of infectious diseases.

## Introduction

The novel coronavirus (COVID-19) can cause severe human respiratory syndrome and is highly infectious^1^. The global COVID-19 pandemic has been going on over three years and caused a global public health crisis, although it was recently declared that the COVID-19 was no longer constitutes a public health emergency of international concern^2^. Up to 2023/06, over 760 million coronavirus cases were confirmed and over 6.9 million deaths were reported^3^. COVID-19 vaccine has also been implemented to prevent and control the pandemic since early 2021^4^, and many governments all around world tried to promote vaccination rate; however, the global vaccination coverage of COVID-19 is lagging^5,6^ and new novel coronavirus variants are continuously emerging^7,8^. Even worse is that, nowadays, it is not yet possible to foresee when this pandemic will end^7–10^ under the situation that we have obtained abundant information about the novel coronavirus in comparison to that at the beginning of this pandemic.

Compared to many countries and regions, relatively strict NPIs have been implemented by Chinese government in 2020. These NPIs mainly include city lockdown, traffic restriction, quarantine, keeping social distance, wearing mask, tracking health QR code, and so on^11^. Except for city lockdown which was usually applied when seriously regional or local events occurred^12^, other measures were regularly implemented for daily prevention and control. In 2021, Chinese government regularly and gradually raised COVID-19 vaccination rate, by companying with rational relaxations on NPIs. The COVID-19 pandemic across Chinese mainland was thus gradually stabilized at a low level before the emergence of Omicron variant; however, regional or local events were sporadically emerged. Therefore, studies on the COVID-19 pandemic (including both confirmed imported and confirmed domestic cases) across Chinese mainland were constantly conducted.

Studies on spatiotemporal characteristics of confirmed domestic cases across Chinese mainland from different perspectives rapidly increased after the outbreak of COVID-19 pandemic (e.g.^13–16^). Later, studies on confirmed imported cases from foreign countries increased when the risk to domestic cases caused by imported cases gradually became evident^17,18^. These studies were mainly motivated by finding out the socio-demographic characteristics, clinical characteristics, and spatiotemporal distributions of confirmed imported cases (e.g.^19–23^). These efforts improved our understanding of the spatiotemporal features of confirmed cases and helped us make reasonable decisions on the selection of prevention and control measures. Nevertheless, researches mentioned above still have some shortcomings. Firstly, even though global cases play an important role in dynamics of imported cases, the relationship between confirmed imported cases and confirmed global cases is not yet evaluated and the change of this relationship between 2020 and 2021 in the same time period is also missed. Secondly, the data employed in studies mentioned above were not enough to reach a comparison on the changes of spatiotemporal distribution characteristics of both confirmed domestic cases and confirmed imported cases (employed data for confirmed domestic cases as of 2020/10^15^, and employed data for confirmed imported cases as of 2021/03/22^23^).

Therefore, to fulfill the gap mentioned above, this paper collected the confirmed cases data across Chinese mainland as well as confirmed global cases during the same period both in 2020 and 2021, analyzed the relationship between confirmed imported cases and confirmed global cases, and conducted a preliminary data comparison on the spatiotemporal distribution of confirmed cases in view of the variation in prevention and control measures during the identical period between the two years. It hopes that findings obtained here could enrich our practical experience about the prevention and control of the COVID-19 pandemic.

## Materials and methods

The confirmed case data during the COVID-19 pandemic across Chinese mainland were gathered from National Health Commission, China^24^ and the provincial health commissions (total 31 provincial administrative regions) across Chinese mainland. The ‘Chinese mainland’ means that the data of Hongkong (China), Macao (China) and Taiwan (China) were not included. The global confirmed case data were gathered from WHO Coronavirus (COVID-19) Dashboard^25^. The gathered data were reported from 2020/04/01 to 2020/08/31 and from 2021/04/01 to 2021/08/31. There were several reasons for selecting these time periods. The first reason is to avoid the effect of novel coronavirus Omicron variant^26^. The epidemiological features in both infectivity and lethality of this variant are different from previous variants^27,28^. For example, the Omicron variant had higher transmissibility but usually caused lower risk of hospitalization and mortality^28^. The second one is to avoid the effect of national holidays, i.e., Mid Autumn Festival and National Day, which usually caused vast population flow^29^. It suggested that vast population flow during national holidays significantly affected the spatiotemporal distribution of COVID-19 in China^29^. Finally, formal and extensive implementation of COVID-19 vaccine across Chinese mainland started from 2021/03^11^. To reach quantitative analyses, curve fits for some raw data were conducted by using the Origin software^30^. Moreover, the confirmed global cases posted on the web site may change because of officially declared modification, with the advance of time. But, the modification usually referred to a small amount of cases, which suggests that the main findings in this study would not be affected.

To better evaluate the correlation between confirmed imported cases and confirmed domestic cases across Chinese mainland, the information about the day (*D*_*1*_) on which the first confirmed imported cases were reported, the total confirmed imported cases (*N*_*1*_) as of 2021/08/31, the total confirmed domestic cases (*N*_*21*_) on the day of *D*_*1*_, and the total confirmed domestic cases (*N*_*22*_) as of 2021/08/31 for each provincial administrative region were collected. The increment of confirmed domestic cases in a provincial administrative region was thus defined as *N*_*22*_*–N*_*21*_.

To quantitatively analyze and compare the spatiotemporal variations of distribution patterns of the confirmed cases (both domestic and imported cases) from provincial level to national level across Chinese mainland, several steps were made. (1) Confirmed cases at national level were sorted by month. (2) The longitude and the latitude of the capital of each provincial administrative region (see Supplementary file for details) were used to represent the location of each administrative region. Then, these locations were classified into 5 categories along both the longitude (i.e., longitude ≤ 105°, 105° < longitude ≤ 110°, 110° < longitude ≤ 115°, 115° < longitude ≤ 120°, and longitude > 120°) and the latitude (i.e., 20° < latitude ≤ 25°, 25° < latitude ≤ 30°, 30° < latitude ≤ 35°, 35° < latitude ≤ 40°, and latitude > 40°). (3) Confirmed cases at national level were classified by longitude (or latitude) category. (4) Confirmed cases at each longitude (or latitude) category were classified by month. (5) Coefficient of variations of classified confirmed cases were calculated. Suppose *X*_*i*_ and $\overline{X }$ are the value of a data point and the mean of all data (*n* points) in one kind of classification, coefficient of variation was then defined as $R={\sigma }_{X}/\overline{X }=\sqrt{{\sum }_{1}^{n}{({X}_{i}/\overline{X }-1)}^{2}/(n-1)}$. So, a higher value of R indicates a higher scatter of data points, which means a higher heterogeneity of confirmed case distributions. In the following, “*R*_*Lat, Imp*_”, “*R*_*Lat, Dom*_”, “*R*_*Lon, Imp*_”, “*R*_*Lon, Dom*_”, “*R*_*Tem, Imp*_”, and “*R*_*Tem, Dom*_” represent coefficient of variations of all data points in latitude classification for imported cases, in latitude classification for domestic cases, in longitude classification for imported cases, in longitude classification for domestic cases, in temporal classification for imported cases, and in temporal classification for domestic cases, respectively. (6) Comparisons on monthly changes of “*R*_*Lat, Imp*_”, “*R*_*Lat, Dom*_”, “*R*_*Lon, Imp*_”, and “*R*_*Lon, Dom*_” and latitudinal (or longitudinal) changes of “*R*_*Tem, Imp*_” and “*R*_*Tem, Dom*_” between 2020/04–2020/08 and 2021/04–2021/08 were conducted.

## Results

### The correlation between confirmed imported cases and confirmed global cases

**Figure 1:**
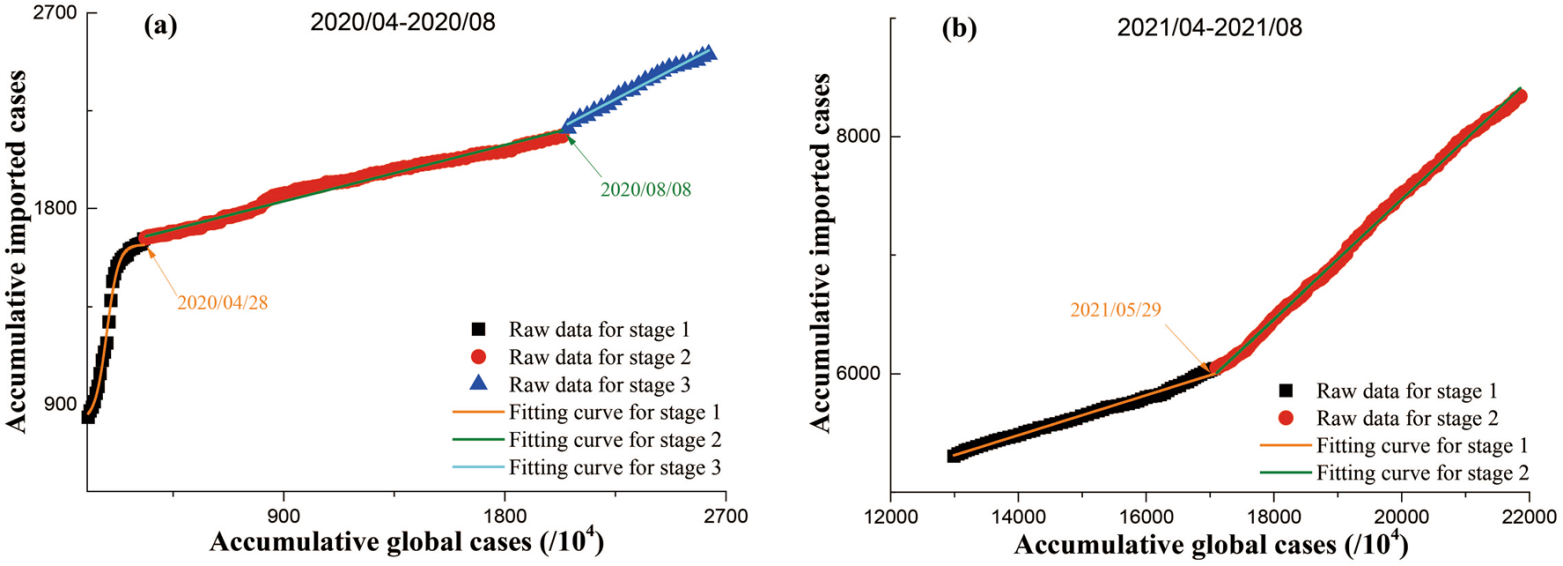
The correlation between accumulative global cases and accumulative imported cases. (**a**) 2020/04–2020/08, (**b**) 2021/04–2021/08. Scatters are raw data; lines are fitting curves. The values of Adj. R^2^ for all fitting curves are larger than 0.99.

It was found from Fig. 1 that, accumulative number of confirmed imported cases both during 2020/04–2020/08 and during 2021/04–2021/08 showed positive correlation with accumulative number of confirmed global cases; however, the correlation between accumulative number of confirmed imported cases and accumulative number of confirmed global cases in 2020 was either qualitatively or quantitatively different from that in 2021. As shown in Fig. 1a, the increase of accumulative imported cases with accumulative global cases could roughly be divided into three stages during 2020/04–2020/08. Further quantitative analysis suggested that the sharp increase of accumulative imported cases with accumulative global cases in stage 1 during 2020/04–2020/08 could be expressed by a dose–response curve, i.e., ${N}_{I20,s1}=826.629+806.9/\{1+{10}^{[0.01842\times (179.683-{N}_{G20,s1}/{10}^{4})]}\}$(R^2^ > 0.99). The variations of accumulative imported cases with accumulative global cases in both stage 2 and stage 3 during 2020/04–2020/08 could be expressed by linear curves, i.e., ${N}_{I20,s2}=1574.87+0.2872\times {N}_{G20,s2}/{10}^{4}$) (R^2^ > 0.99) and ${N}_{I20,s3}=975.98+0.5899\times {N}_{G20,s3}/{10}^{4}$) (R^2^ > 0.99). In contrast, the increase of accumulative imported cases with accumulative global cases could roughly be divided into two stages during 2021/04–2021/08 (Fig. 1b). Further quantitative analysis suggested that the variations of accumulative imported cases with accumulative global cases in both stage 1 and stage 2 during 2021/04–2021/08 could be expressed by linear curves, i.e., ${{N}_{I21,s1}=3120.2+0.1687\times {N}_{G21,s1}/10}^{4}$) (R^2^ > 0.99) and ${N}_{I21,s2}=-2635.44+0.5053\times {N}_{G21,s2}/{10}^{4}$) (R^2^ > 0.99).

### Relationship between confirmed domestic cases and confirmed imported cases as of 2021/08/31

**Figure 2:**
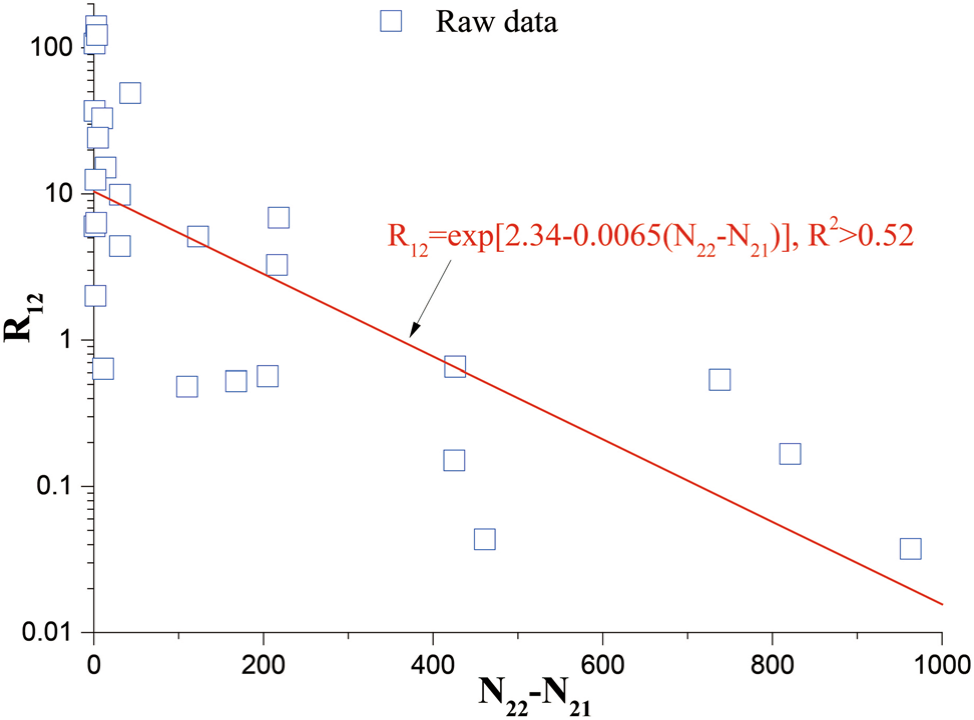
The relationship between *R*_*12*_ and *N*_*22*_–*N*_*21*_. Open scatters are raw data; the solid line is the fitting curve. The value of Adj. R^2^ for the fitting curve is larger than 0.52.

Gathered data (Table 1) revealed that confirmed imported cases were widely distributed across Chinese mainland, except for three provincial administrative regions (i.e., Xinjiang, Qinghai and Tibet). As of 2021/08/31, Guizhou confirmed the least imported case (only 1 case), while Shanghai confirmed the most imported cases (2102 cases). For confirmed domestic cases, all provincial regions have reported confirmed cases (Table 1). As of 2021/08/31, cumulative confirmed domestic cases reached up to 86,558 across Chinese mainland, Tibet had the least cumulative confirmed domestic case (only 1 case), and Hubei had the most cumulative confirmed domestic cases (68,226 cases). Therefore, high (or low) number of cumulative confirmed domestic cases did not mean high (or low) number of cumulative confirmed imported cases in a place. It revealed that the increments (*N*_*22*_–*N*_*21*_) of confirmed domestic cases ranged over three orders of magnitude (minimum value of* N*_*22*_*–N*_*21*_ is 0, and maximum value of* N*_*22*_*–N*_*21*_ is 963) among 31 provincial administrative regions. The* N*_*22*_–*N*_*21*_ showed very weak relation with both cumulative imported cases and cumulative domestic cases. However, the ratio *R*_*12*_ [which is defined as *N*_*1*_/(*N*_*22*_–*N*_*21*_)] showed a decreasing trend with *N*_*22*_*–N*_*21*_ (Fig. 2). Further analysis suggested that the ratio *R*_*12*_ decreased exponentially with *N*_*22*_*–N*_*21*_; and the fitting curve for the collected data (no suitable data of Guizhou, Xinjiang, Qinghai and Tibet were employed) was *R*_*12*_ = Exp[2.34–0.0065(*N*_*22*_–*N*_*21*_)] (R^2^ > 0.52) (Fig. 2). This suggested that the increase of confirmed imported cases may be inhibited in the place (at least for a provincial region in this study) where high increase of confirmed domestic cases existed across Chinese mainland.

**Table 1:** General information about the COVID-19 pandemic across Chinese mainland as of 2021/08/31. *D*_*1*_, *N*_*1*_, *N*_*21*_, *N*_*22*_ and *R*_*12*_ are the day on which the first imported cases in a provincial administrative region was confirmed, the total imported cases as of 2021/08/31, the total domestic cases as of the day *D*_*1*_, the total domestic cases as of 2021/08/31, and the ratio of *N*_*1*_/(*N*_*22*_–*N*_*21*_), respectively.

	Location	*D*_*1*_	*N*_*1*_	*N*_*21*_	*N*_*22*_	*N*_*22*_–*N*_*21*_	*R*_*12*_
1	Ningxia	2020/02/26	4	71	73	2	2.00
2	Beijing	2020/02/29	280	411	837	426	0.66
3	Guangdong	2020/03/01	1498	1349	1567	218	6.87
4	Zhejiang	2020/03/01	212	1205	1219	14	15.14
5	Gansu	2020/03/05	107	91	92	1	107.00
6	Shanghai	2020/03/05	2102	338	381	43	48.88
7	Shandong	2020/03/10	136	758	789	31	4.39
8	Henan	2020/03/11	88	1272	1440	168	0.52
9	Yunnan	2020/03/15	703	174	390	216	3.25
10	Guangxi	2020/03/16	37	252	253	1	37.00
11	Shaanxi	2020/03/16	421	245	248	3	140.33
12	Sichuan	2020/03/17	628	439	562	123	5.11
13	Heilongjiang	2020/03/18	395	482	1220	738	0.54
14	Fujian	2020/03/19	489	296	300	4	122.25
15	Liaoning	2020/03/19	116	125	330	205	0.57
16	Tianjin	2020/03/19	329	136	146	10	32.90
17	Hebei	2020/03/21	36	318	1281	963	0.04
18	Jiangsu	2020/03/21	137	631	1452	821	0.17
19	Jiangxi	2020/03/21	6	930	931	1	6.00
20	Chongqing	2020/03/22	25	576	578	2	12.50
21	Shanxi	2020/03/23	121	133	138	5	24.20
22	Jilin	2020/03/24	20	93	554	461	0.040
23	Inner Mongolia	2020/03/24	306	75	106	31	9.87
24	Guizhou	2020/03/28	1	146	146	0	INF
25	Hubei	2020/03/31	64	67,801	68,226	425	0.15
26	Hunan	2020/04/01	53	1018	1128	110	0.48
27	Anhui	2020/04/08	7	990	1001	11	0.64
28	Hainan	2020/05/15	19	168	171	3	6.33
29	Xinjiang	NAN	0	NAN	980	NAN	NAN
30	Qinghai	NAN	0	NAN	18	NAN	NAN
31	Tibet	NAN	0	NAN	1	NAN	NAN

‘INF’ suggests an infinite value because of *N*_*22*_–*N*_*21*_ = 0. ‘NAN’ indicates no imported cases.

### Temporal distributions of confirmed imported cases and confirmed domestic cases during 2020/04–2020/08 and 2021/04–2021/08

**Figure 3:**
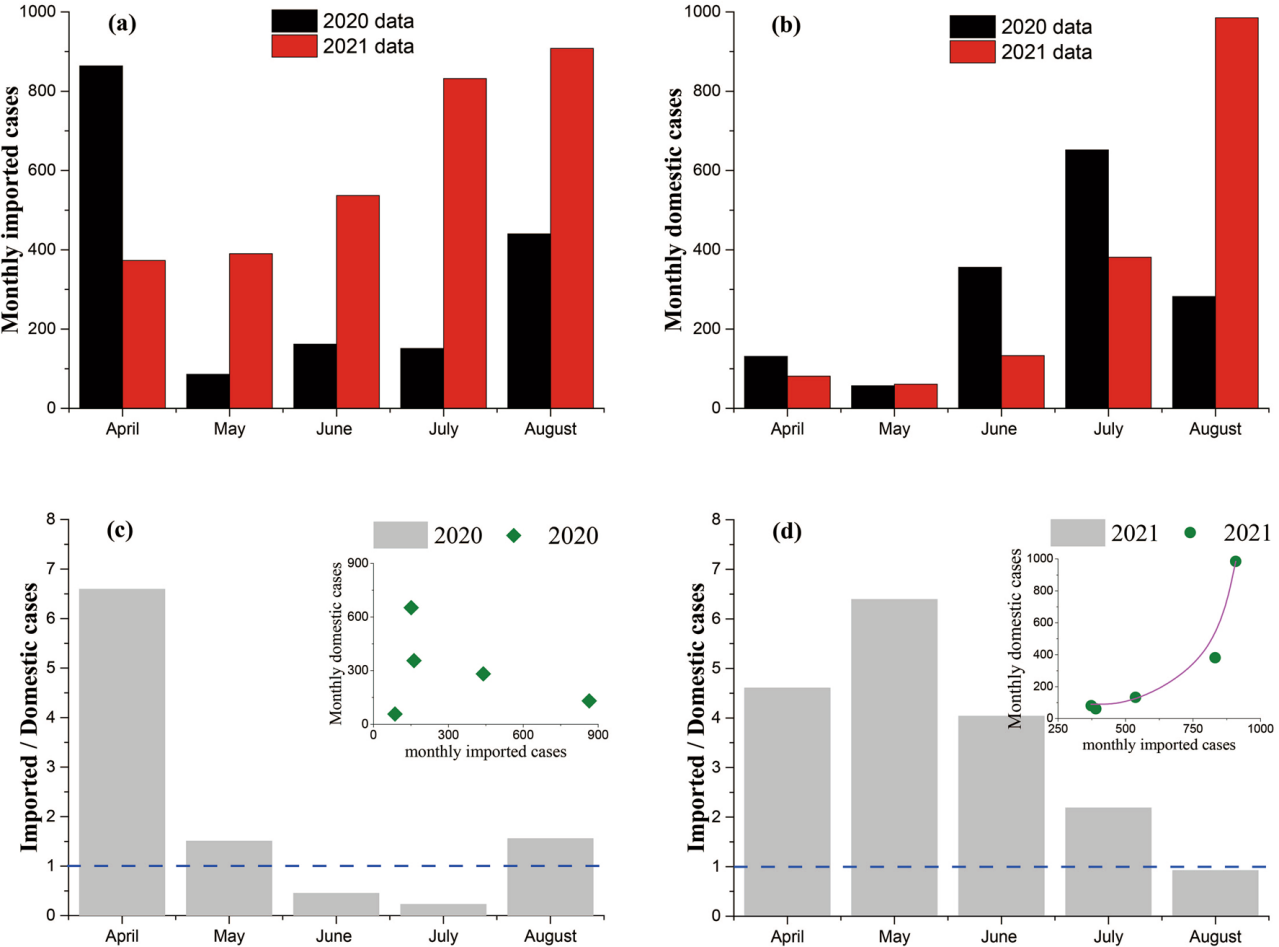
The monthly imported cases and domestic cases during 2020/04–2020/08 and 2021/04–2021/08 across Chinese mainland. Panels (**a**) and (**b**): monthly imported cases and monthly domestic cases during the same period in both years, respectively, panels (**c**) and (**d**): the ratio of imported cases versus domestic cases during 2020/04–2020/08 and 2021/04–2021/08, respectively. Correlation between monthly imported cases and monthly domestic cases is shown in inserts in panels (**c**) and (**d**). Solid line in panel (**d**) is fitting curve, i.e., Y = 89.567 + 0.00148exp(0.01466X) (R^2^ > 0.99), *Y* monthly domestic cases, *X* monthly imported cases.

Generally, both monthly confirmed imported cases and monthly confirmed domestic cases showed remarkable variations with the advance of time (Fig. 3). During 2020/04–2020/08, total 1703 imported cases were confirmed (Fig. 3a), the most confirmed imported cases (864 cases) were reported in April, and the least confirmed imported cases (86 cases) were reported in May. During 2021/04–2021/08, total 3040 imported cases were confirmed (Fig. 3a), the most confirmed imported cases (908 cases) were reported in August, and the least confirmed imported cases (373 cases) were reported in April. From April to August, monthly confirmed imported cases decreased firstly and then increased in 2020, while monthly confirmed imported cases monotonously increased in 2021 (Fig. 3a). During 2020/04–2020/08, total 1478 domestic cases were confirmed (Fig. 3b), the most confirmed domestic cases (652 cases) were reported in July, and the least confirmed domestic cases (57 cases) were reported in May. During 2021/04–2021/08, total 1641 domestic cases were confirmed (Fig. 3b), the most confirmed domestic cases (985 cases) were reported in August, and the least confirmed domestic cases (61 cases) were reported in May. From April to August, monthly confirmed domestic cases increased firstly and then decreased in 2020, while monthly confirmed domestic cases almost monotonously increased in 2021(Fig. 3b).

Besides, the temporal variation of the ratio of monthly confirmed imported cases versus monthly confirmed domestic cases during 2020/04–2020/08 (Fig. 3c) was different from that during 2021/04–2021/08 (Fig. 3d). During 2020/04–2020/08, the ratios in both June and July were smaller than 1.0, the highest ratio occurred in April while the lowest ratio occurred in July. During 2021/04–2021/08, only the ratio in August is smaller than 1.0, the highest ratio occurred in May while the lowest ratio occurred in August. From April to August, the ratios decreased firstly and then increased a little in 2020 (Fig. 3c), while the ratios increased firstly and then decreased gradually in 2021(Fig. 3d). Furthermore, there was no evident correlation between monthly confirmed imported cases and monthly confirmed domestic cases during 2020/04–2020/08 (Insert in Fig. 3c); while monthly confirmed domestic cases during 2021/04–2021/08 increased with monthly confirmed imported cases (Insert in Fig. 3d). Data analysis suggested monthly confirmed domestic cases during 2021/04–2021/08 increased in an exponential form, i.e., Y = 89.567 + 0.00148exp(0.01466X) (R^2^ > 0.99), where Y represented monthly confirmed domestic cases, and X represented monthly confirmed imported cases.

### Spatial distributions of confirmed imported cases and confirmed domestic cases during 2020/04–2020/08 and 2021/04–2021/08

**Figure 4:**
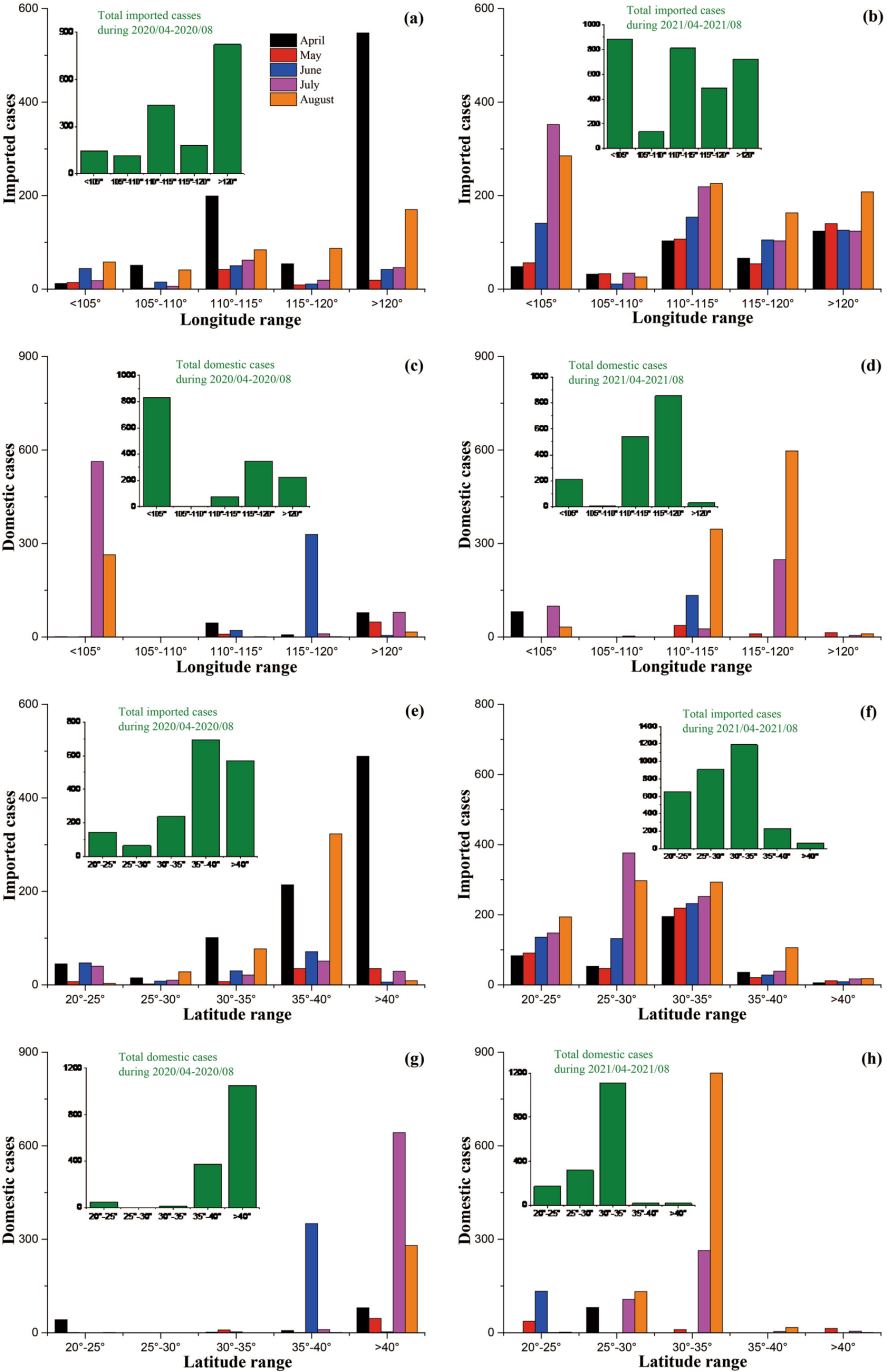
The spatial distributions of total imported and domestic cases during 2020/04–2020/08 and 2021/04–2021/08 across Chinese mainland. Panels (**a**–**d**) along with longitude, panels (**e**–**h**) along with latitude.

The spatial distributions (along with longitude or latitude) of both confirmed imported cases and confirmed domestic cases varied between the two years (Fig. 4). The confirmed imported cases were mainly distributed in the regions of 110° < longitude ≤ 115° and longitude > 120° during 2020/04–2020/08 (Fig. 4a), while widely distributed throughout the longitude range during 2021/04–2021/08 (Fig. 4b). The confirmed imported cases were mainly distributed in the regions of 35° < latitude ≤ 40° and latitude > 40° during 2020/04–2020/08 (Fig. 4e), while mainly distributed in the regions of 20° < latitude ≤ 35° during 2021/04–2021/08 (Fig. 4f). The confirmed domestic cases were mainly distributed in the region of longitude < 105° during 2020/04–2020/08 (Fig. 4c), while mainly distributed in the regions of 110° < longitude ≤ 120° during 2021/04–2021/08 (Fig. 4d). The confirmed domestic cases were mainly distributed in the region of latitude > 40° during 2020/04–2020/08 (Fig. 4g), while mainly distributed in the regions of 30° < latitude ≤ 35° during 2021/04–2021/08 (Fig. 4h). Moreover, it revealed that the temporal change of the spatial distributions of both confirmed imported cases and confirmed domestic cases were relatively frequent (Fig. 4).

**Figure 5:**
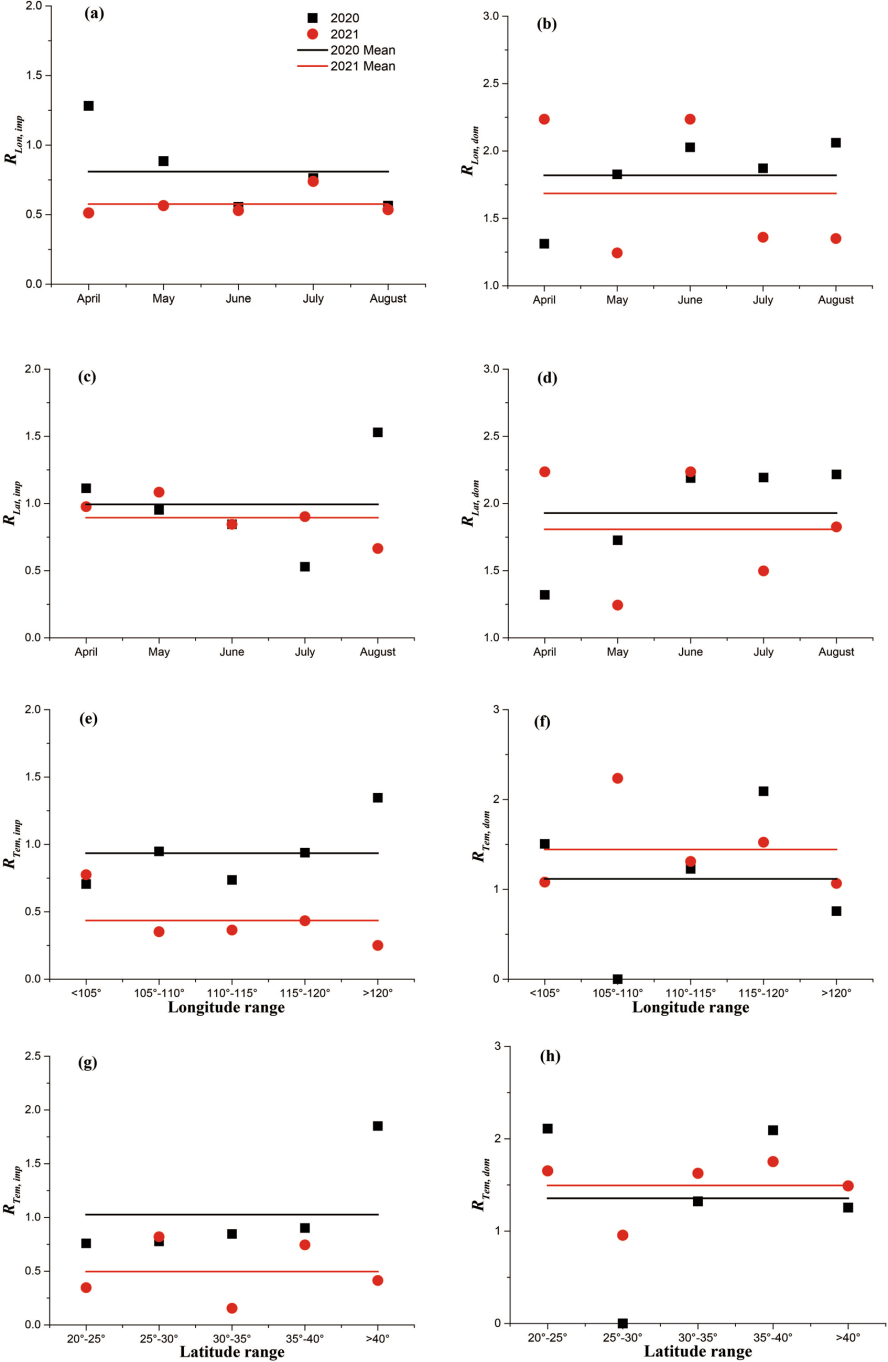
Variations of coefficient of variations with month (**a**–**d**), longitude (**e**, **f**), and latitude (**g**, **h**) during 2020/04–2020/08 and 2021/04–2021/08 across Chinese mainland.

Quantitative analyses suggested the dynamics of spatiotemporal distribution for confirmed imported cases were different from those for confirmed domestic cases (Fig. 5). As for confirmed imported cases, either monthly changes of coefficient of variations, *R*_*Lon, Imp*_ (or *R*_*Lat, Imp*_), for longitude (or latitude) classification (Fig. 5a and Fig. 5c) or longitudinal (or latitudinal) changes of coefficient of variations, *R*_*Tem, Imp*_, for temporal classification (Fig. 5e,g) were observed both during 2020/04–2020/08 and during 2021/04–2021/08; however, means of monthly changed *R*_*Lon, Imp*_, monthly changed *R*_*Lat, Imp*_, longitudinally changed *R*_*Tem, Imp*_, and latitudinally changed *R*_*Tem, Imp*_ during 2020/04–2020/08 were higher than those during 2021/04–2021/08. This suggested that spatiotemporal distribution of confirmed imported cases during 2021/04–2021/08 was overall more homogeneous than that during 2020/04–2020/08. As for confirmed domestic cases, fluctuations of coefficient of variations still existed among all focused situations; however, means of monthly changed *R*_*Lon, Imp*_ and monthly changed *R*_*Lat, Imp*_ during 2020/04–2020/08 were higher than those during 2021/04–2021/08 (Fig. 5b,d), while means of longitudinally changed *R*_*Tem, Imp*_ and latitudinally changed *R*_*Tem, Imp*_ during 2020/04–2020/08 were lower than those during 2021/04–2021/08 (Fig. 5f,h). This suggested that spatial distribution of confirmed domestic cases during 2021/04–2021/08 was more homogeneous than that during 2020/04–2020/08, while temporal distribution of confirmed domestic cases during 2021/04–2021/08 was more heterogeneous than that during 2020/04–2020/08. Besides, coefficient of variations of confirmed domestic cases both during 2020/04–2020/08 and during 2021/04–2021/08 were higher than those of confirmed imported cases, which suggested that spatiotemporal distribution of confirmed domestic cases was more heterogeneous than that of confirmed imported cases.

## Discussion

Globally public health crisis caused by the COVID-19 pandemic could not be tided over in a short time^31^, because novel coronavirus variants are continuously emerging^32^. However, international travel and trade in goods had to be restarted because of basic demands for our lives and the increase of pressure in economic growth. It is thus inevitable that confirmed cases would be imported from foreign countries once international travel and trade in goods exist because of difference in policies of COVID-19 prevention and control among different countries. And, it is easy to understand that these different policies could cause the difference in the temporal changes between confirmed imported cases and confirmed global cases. The positive (most of time linear) relationship between cumulative imported cases and cumulative global cases (Fig. 1) demonstrated that global cases indeed could affect imported cases. And, the change in the increase rates of cumulative imported cases with cumulative global cases (see fitting curves above) in the same period between 2020 and 2021 reflects the effectiveness of the adjustment of the Chinese government’s policies of COVID-19 prevention and control responding to the development of COVID-19 pandemic at home and abroad. It should be noted that both the division of different stages and the selection of fitting curves here were carried out to roughly but simply address the main difference in the variations of imported cases along with global cases. So, only two or three stages were divided and simple curve forms (exponential or linear) were selected. However, the stage division and the curve selection are not unique. Typically, the more stages are divided, the more diverse the fitting curves become, depending on specific purposes or situations. For example, the evolution of the COVID-19 epidemic in the places across Chinese mainland (except for Hubei province) during 2020/01/20–2020/03/12 was divided into five stages^13^.

Although imported cases did play an important in the evolution of domestic cases^33,34^, high number of confirmed imported cases did not mean high number of confirmed domestic cases. In contrast, confirmed imported cases may be inhibited statistically in the place where high increase of confirmed domestic cases existed across Chinese mainland (Fig. 2 and Table 1). This could be explained from both personal and government perspectives. When the number of confirmed domestic cases (which could be easily found from various media sources both in China and worldwide) in an original destination increases, the destination would be regarded as the place of high infection risk. Thus, individuals, who planned to go to the destination, would turn to select other places as ports of entry because of human beings' nature of seeking profit and avoiding harm. At the same time, the government would timely tighten prevention and control policies (e.g., decreasing both population inflow and outflow) until the epidemic in the destination was well controlled. So, the importation of the infection risk would be kept at a low level.

In the absence of COVID-19 vaccine (corresponding to 2020/04–2020/08), non-pharmacological interventions (NPIs) were the most important measures to prevent and control COVID-19 epidemic^12^. Meanwhile, people have not received enough information and practical experience about COVID-19 epidemic. So, people showed sufficiently tolerant attitude towards the relatively strict NPIs and regularly followed these NPIs, which resulted in rapid control of national COVID-19 epidemic and low level of local COVID-19 outbreaks. Therefore, during 2020/04–2020/08, Chinese mainland was thought to be the place that had lower infection risk than many other places worldwide. Many people (mainly overseas Chinese) traveled to Chinese mainland, importing confirmed cases from abroad. Particularly, confirmed imported cases in 2020/04, which accounted for 50.73% of total confirmed imported cases during 2020/04–2020/08 (Fig. 3a), were six times higher than confirmed domestic cases in 2020/04 (Fig. 3c). Such high ratio of imported cases versus domestic cases and the impact of imported cases on domestic cases make Chinese government tighten international travel restrictions (e.g., “circuit breaker” mechanism for airlines). The confirmed imported cases during 2020/05–2020/08 (even though cases increased slowly with time due to the change of prevention and control policies) were thus largely reduced (Fig. 3a), and the ratio of imported cases versus domestic cases kept lower than 1.5 during 2020/05–2020/08 (Fig. 3c). By limiting the effect of imported cases, the temporal change of confirmed domestic cases could be mainly caused by the nationwide activities, for examples, summer vacation and summer travel. As recent study^35^ pointed out, the temperature and travel flux made important contribution to the infection and consequently the infection-induced death. In fact, many Chinese families would like to take their children on vacation trips in summer, increasing inter-city travel flux nationwide. The high temperature in summer (particularly under current situation that extreme weather conditions become frequent) would prompt people to take their families to open or indoor swimming pools, increasing local travel flux within a city. Confirmed domestic cases thereby rebounded during 2020/06–2020/08 (the highest value in 2020/07) compared to those during 2020/04–2020/05 (Fig. 3b), in accord with previous study in which the prevalence of the epidemic was found to be significantly correlated with temperature^13^. Therefore, monthly confirmed domestic cases showed weak dependence on monthly confirmed imported cases (Insert in Fig. 3c).

The advent of COVID-19 vaccines (corresponding to 2021/04–2021/08) boosted the confidences of both government and ordinary people. Chinese government properly relaxed NPIs with promoting vaccination rate^24^. Some people gradually lost their tolerance on the restrictions of NPIs, particularly on travel restrictions. These two aspects lead to the increase of both confirmed imported cases and confirmed domestic cases during 2021/04–2021/08 in comparison to the situation during 2020/04–2020/08 (Figs. 3 and 4). However, different from that confirmed imported cases were comparable to confirmed domestic cases during 2020/04–2020/08, confirmed imported cases became about two times of confirmed domestic cases. This may be because, compared to the contemporaneously serious situation worldwide, the low level of the COVID-19 pandemic made many people believe Chinese mainland was much safer, apart from the slightly loosed international travel restrictions. Another difference in temporal distributions of the COVID-19 epidemic between 2020/04–2020/8 and 2021/04–2021/08 was that both confirmed imported cases and confirmed domestic cases reached the maximum value in August (Fig. 3a,b). And, both monthly confirmed imported cases and monthly confirmed domestic cases generally increased with time, which resulted in an exponential relationship between monthly confirmed imported cases and monthly confirmed domestic cases (Insert in Fig. 3d). This exponential relationship indicated that imported cases were partially contributed to the increase of domestic cases during 2021/04–2021/08. Therefore, the temporal distribution of confirmed domestic cases during 2021/04–2021/08 was more heterogeneous than that during 2020/04–2020/08 (Fig. 5f,h).

In contrast, the spatial distributions of the COVID-19 epidemic across Chinese mainland were more complex. Different from the early stage of the COVID-19 epidemic (before 2020/04), in which the spatial distributions were significantly affected by the epidemic in Hubei province and were concentrated around Hubei^13^, both confirmed imported cases and confirmed domestic cases mentioned here were widely distributed but no longer related to the epidemic in Hubei (Fig. 4). The main reason for the spatial distribution of confirmed imported cases may be the way and the location of entry. People entered Chinese mainland mainly from air ports and land ports on border. The distribution of port locations and the travel restrictions at these ports were thus crucial factors affecting the spatial distributions of confirmed imported cases. For example, many imported cases that entered from the Suifenhe port in Heilongjiang province in China in 2020/04^21^ were confirmed, which leaded to the concentration of imported cases at high longitude and latitude regions during 2020/04–2020/08 (Fig. 4a,e). The travel restrictions at the ports on north border were then quickly tightened to decrease the importation risk. People turned to select the entry ports on southwest boarder (e.g., Guizhou province and Yunnan province) during 2021/04–2021/08, which resulted in that many confirmed imported cases concentrated at low longitude and latitude regions (Fig. 4b,f). However, the main reasons for the spatial distribution of confirmed domestic cases could be various and diverse. The first reason is the route of infection. The common infection ways for domestic cases were infected by confirmed cases, by asymptomatic cases, and by the materials containing COVID-19, respectively^11^. Statistically, the spatially distributed confirmed domestic cases showed very weak dependence on spatially distributed confirmed imported cases (Fig. 4). This suggested that the contribution of confirmed imported cases may be low under the NPIs implemented by Chinese government, although confirmed imported cases indeed would affect confirmed domestic cases^33,34^. The contact transmission (contacting materials containing COVID-19) could be an important contributor for the spatial distribution of confirmed domestic cases. For instance, a local outbreak of COVID-19 epidemic in Beijing during 2020/06/11–2020/07/05 was caused by cold goods^36^. There were total 335 domestic cases confirmed during this local outbreak, which accounted for 22.67% of confirmed domestic cases during 2020/04–2020/08. Besides, the local outbreak in Xinjiang (the original infection route had not been found) contributed to the concentration of confirmed domestic cases at low longitude but high latitude regions during 2020/04–2020/08 (Fig. 4c,g), which also resulted in the higher spatial heterogeneity of confirmed domestic cases during 2020/04–2020/08 than that during 2021/04–2021/08 (Fig. 5b,d). Finally, the advent of COVID-19 vaccines and the relaxed NPIs, which would increase population flux as well as the dispersal of asymptomatic cases, should be one main driver of the difference in spatiotemporal distributions of confirmed domestic cases between 2020 and 2021.

## Supplementary Information

Supplementary Information.

## Data Availability

All data generated or analyzed during this study are included in this published article (and its Supplementary Information Files).
